# Physical, social and societal functioning of children with congenital adrenal hyperplasia (CAH) and their parents, in a Dutch population

**DOI:** 10.1186/1687-9856-2012-2

**Published:** 2012-02-02

**Authors:** Sarita A Sanches, Therese A Wiegers, Barto J Otten, Hedi L Claahsen-van der Grinten

**Affiliations:** 1 Netherlands institute for health services research (NIVEL), P.O. Box 1568, 3500 BN Utrecht, The Netherlands; 2 VU Medical Centre, department of Rehabilitation Medicine, room PK -1 Y 158, De Boelelaan 1118, 1081 HZ, The Netherlands; 3 Department of Paediatric Endocrinology, Radboud University Nijmegen Medical Centre, P O Box 9101, 6500 HB Nijmegen, The Netherlands

**Keywords:** CAH, children, quality of life, social functioning, burden of disease, self-management, participation, Netherlands, parents, comorbidity, preventive measures

## Abstract

**Background:**

Most research concerning congenital adrenal hyperplasia (CAH) and related conditions caused by primary adrenal insufficiency, such as Addison's or Cushing's disease, has focused on medical aspects rather than on patients' quality of life. Therefore, our objective was to investigate the physical, social and societal functioning of children with CAH and their parents in a Dutch population.

**Methods:**

The study is descriptive and cross-sectional. Self-designed questionnaires, based on questionnaires developed in the Netherlands for different patient groups, were sent to parents of children with CAH between 0 and 18 years old. Participants were recruited through the Dutch patient group for Adrenal Disease (NVACP) and six hospitals in the Netherlands. Three different questionnaires were designed for parents: for children aged 0 - 4, aged 4 - 12 and aged 12 - 18. Additionally, a fourth questionnaire was sent to adolescents with CAH aged 12 - 18. Main outcome measures were experienced burden of the condition, self-management and participation in several areas, such as school and leisure time.

**Results:**

A total of 106 parents returned the questionnaire, 12 regarding pre-school children (0-4 years), 63 regarding primary school children (4-12 years), and 32 regarding secondary school children (12-18 years), combined response rate 69.7%. Also, 24 adolescents returned the questionnaire. Children and adolescents with CAH appear to be capable of self-management at a young age. Experienced burden of the condition is low, although children experience several health related problems on a daily basis. Children participate well in school and leisure time. Few children carry a crisis card or emergency injection with them.

**Conclusions:**

Overall, our research shows that, according to their parents, children with CAH experience few negative effects of the condition and that they participate well in several areas such as school and leisure time. However, improvements can be made concerning the measures parents and children must take to prevent an adrenal crisis.

## Background

Congenital Adrenal Hyperplasia (CAH) is an inherited disorder of the adrenal cortex caused by deficiency of enzymes involved in adrenal steroidogenesis, most often a deficiency of 21-hydroxylase [1-4]. This defect results in an impaired production of cortisol and mostly also of aldosterone and an excessive production of adrenal androgens. The clinical picture depends on the degree of the enzymatic block: the most severe form, the classic CAH (almost always apparent at birth) and the mild nonclassic form (mostly diagnosed later in life). In general, CAH has larger implications for females than males. Furthermore, the classical form is subclassified in the salt wasting (SW) and the simple virilising form (SV) [3]. Females with the classic form of CAH are born with ambiguous external genitalia caused by the excessive amount of androgens already in utero [3,5]. In these cases, surgery is often necessary to correct the external genitalia. Treatment of CAH consists of long-term glucocorticoid and mostly also mineralocorticoid substitution [6]. Increased dosages of glucocorticoids are necessary in case of physical stress to prevent life threatening Addisonian (adrenal) crisis. Overtreatment can lead to growth inhibition, excess weight, and several daily health related problems such as sleepiness and abdominal pain. Undertreatment can lead to symptoms of androgen excess such as signs of early puberty, early growth acceleration and reduced final height. In the Netherlands, the prevalence of CAH is about 1 in 12.000. Every year, 15 to 20 infants are newly diagnosed with the disorder [7]. Since 2000, CAH is part of the neonatal screening programme in the Netherlands to prevent life threatening Adrenal crisis.

Studies focussing on quality of life in patients with adrenal diseases are rare. Only recently some studies have been published about the quality of life of adult patients with Addison's disease. Most of these studies have shown a reduced quality of life, especially in adult patients with comorbidity. Furthermore, a recent study [8] found that the objective and subjective health status in adult CAH patients in the United Kingdom was significantly impaired. However, no research has been carried out to study the quality of life of children with primary adrenal insufficiency.

The aim of our study is to investigate the quality of life of children and adolescents with CAH and their parents by studying physical, social and societal functioning of children with CAH and their parents.

## Methods

### Subjects

Parents of children with all types of CAH were recruited through the Dutch patient group Adrenal Disease (NVACP). Additionally, several hospitals in the Netherlands were asked to inform their patients about the research. A total of six hospitals participated in the study, five university hospitals and one general hospital.

### Assessments

Self designed questionnaires, based on questionnaires developed in the Netherlands for different patient groups, were used to measure the following sub-concepts: social demographics (i.e. sex, age, parents' education, country of origin), characteristics of the condition (i.e. CAH subtype, comorbidity, CAH-medication use), experienced burden of the condition (physical and mental problems, constraints, adrenal crisis), self management (lifestyle, social support, general medication use) and participation in several areas such as school and leisure time. The questions were derived from the Second National Survey of General Practice (DNSGP-2) [9], a Dutch study concerning quality of life of (adult) patients with Addison's disease, Cushing's disease and CAH [10] and a Dutch study concerning young adults with chronic digestive disorders [11].

Three different questionnaires were developed for parents of different age groups. A questionnaire for parents of children aged 0 - 4, 4 - 12 and 12 - 18. This distinction was based on the Dutch schooling system. Children aged 0 - 4 do not go to school yet, children aged 4 - 12 receive primary education and children aged 12 - 18 receive secondary education. The questionnaires were largely the same, except for some age-specific questions, for instance, about school, work and going out. Adolescents of 12 - 18 years were also invited to fill out a questionnaire themselves [12].

### Study design

In this study, quality of life is defined according to the sub-concepts used by Heijmans et al [10] in a Dutch report about the impact of Addison's disease, Cushing's disease and CAH on the daily life of adult patients. The sample of CAH patients in that study was too small to draw conclusions from and children were excluded.

An exploratory design was used to describe the several sub-concepts (see assessments).

Parents and adolescents had the possibility to fill out a paper version or an online version of the questionnaire. For the online version, they received a link to the questionnaire by email. A first reminder was sent after 2-3 weeks. If needed, a second reminder was sent again 2-3 weeks later.

### Ethical approval

Ethical approval is not required for this kind of study in the Netherlands.

### Analysis

Due to the division in three separate age groups, the number of subjects in each age group was relatively small, which hampered the use of statistical models. Therefore, the results are mainly descriptive. Each concept was described for each group and groups were compared to study possible differences on the various concepts. Six (for age group 0-4) or seven (for both other age groups) questions have been combined in order to create the scale 'Parents' experiences'. Eight questions were combined to create the scale 'Difficulties'. The reliability of these scales is expressed in Cronbach's alpha. Where possible, comparisons were made with data from the second Dutch National Survey of General Practice (DNSGP-2). DNSGP-2 is a Dutch nationwide study, containing representative information on morbidity in the population, use of health services at patient level, health determinants and sociodemographic characteristics [9]. DNSGP-2 contains data about approximately 50.000 children in the Netherlands, which makes it a very powerful dataset for comparison. All analyses were carried out with Stata 10.

## Results

### Response

250 invitations were sent to parents of children with CAH with information about the study and 152 parents enrolled and received a questionnaire. Of those questionnaires 106 were returned fully completed (response rate of 69.7%). Twelve questionnaires were returned by parents of children of 0-4 years, 62 for children of 4-12 years and 32 for adolescents 12-18 years of age. Adolescents themselves returned 24 questionnaires.

### Sample characteristics

#### Parent questionnaires

Sample characteristics are listed in Table 1. The groups 0-4 and 4-12 consisted of slightly more boys than girls. This was reversed in the group 12-18. The age distribution within the groups was skewed. Children under the age of 1 year were not represented in the youngest age group. The middle age group was relatively old with 69% of children being over 7 years old. On the other hand, the oldest age group was relatively young with almost 63% of children being under 15 years old. The mean age of children in the group 0-4 was 2.4 years, in the middle age group 8.5 years and in the oldest age group 15.0 years. Most children (75%) had the salt wasting type of CAH, only 5% of children had the nonclassic type of CAH. In all age groups, boys were more often diagnosed as salt wasters than girls (boys ≥ 80% SW, girls 50-75% SW). The time from the first symptoms until the definitive diagnosis was shorter for younger children than for older children. Furthermore, younger children experienced fewer health related problems before the time of diagnosis than older children.

**Table 1:** Sample characteristics and characteristics of the condition from parent questionnaires

	0-4 year		4-12 year		12-18 year		Total	
	
	n	*(%)*	n	*(%)*	n	*(%)*	n	*(%)*
Total	12	*(100)*	62	*(100)*	32	*(100)*	106	*(100)*
**Sex**								
Male	7	*(58)*	34	*(55)*	15	*(47)*	56	*(53)*
Female	5	*(42)*	28	*(45)*	17	*(53)*	50	*(47)*
**CAH subtype**								
Salt wasting form	9	*(75)*	50	*(81)*	21	*(66)*	80	*(75)*
Simple virilizing form	3	*(25)*	9	*(14)*	8	*(25)*	20	*(19)*
Non-classical form	0	*-*	3	*(5)*	2	*(6)*	5	*(5)*
Unknown	0	*-*	0	*-*	1	*(3)*	1	*(1)*
**Diagnosis**								
Within 1 week after birth	11	*(92)*	45	*(73)*	12	*(38)*	68	*(64)*
**Health related problems before diagnosis**								
Yes	6	*(50)*	42	*(68)*	24	*(75)*	72	*(70)*
**Complications at birth**								
Yes	5	*(42)*	19	*(31)*	9	*(28)*	33	*(31)*
**Reconstructive surgery (girls)**	n = 5		n = 28		n = 17		n = 50	
Yes	3	*(60)*	15	*(54)*	6	*(35)*	24	*(48)*

#### Adolescent questionnaires

Sample characteristics are listed in Table 2. 24 questionnaires were returned (62.5% girls). 79% indicated to have reached puberty. 79.2% had salt wasting CAH, 8.3% SV CAH and 1 person (4.2%) had the nonclassic form of CAH. 33% of adolescents reported to experience no daily health related problems, 17% experienced one, 13% two, 4% three, and 33% more than four daily health related problems.

**Table 2:** Sample characteristics and characteristics of the condition from adolescent questionnaires

	Adolescents	
	
	n	*(%)*
Total	24	*(100)*
**Sex**		
Male	9	*(37.5)*
Female	14	*(62.5)*
**Age**		
12 - 15	14	*(62.5)*
15 - 18	9	*(37.5)*
**Reached puberty**		
Yes	19	*(79)*
No	5	*(21)*
**CAH subtype**		
Salt wasting form	19	*(79)*
Simple virilizing form	2	*(8)*
Non-classical form	1	*(4)*
Unknown	2	*(8)*
**Number of daily health-related problems**		
0	8	*(33)*
1	4	*(17)*
2	3	*(13)*
3	1	*(4)*
≥ 4	8	*(33)*

#### Health related problems at diagnosis

Health related problems often mentioned by parents in all age groups are weight loss, severe somnolence, accelerated growth/development, inability to retain fluids and lack of appetite. About 31% of the children had CAH related complications in the neonatal period. Genital anomalies occurred most often, dehydration due to loss of salt was second. Girls in the younger age groups more often underwent reconstructive surgery than in the older group. One parent of a child aged 0-4 years was dissatisfied with the results of the operation, whereas all other parents of operated children (n = 24) were satisfied with the results of the operation.

#### Current health related problems and comorbidity

Table 3 shows the number of daily health related problems and the incidence of chronic conditions beside CAH such as asthma or eczema in the sample from the parent questionnaires compared to data from DNSGP-2. Although chronic conditions in addition to CAH were rare in the study population, 71% of the children experienced daily health related problems in the two weeks preceding the questionnaire. The daily health related problems that occurred most often in children with CAH were excessive sweating, mood swings, lack of energy, excitability, fever, sleepiness, crying and pain in the legs and abdomen. However, in all age groups, children with CAH seem to have less daily health related problems than their peers in DNSGP-2 but this finding was not tested for significance. Only children aged 4-12 seem to have more daily health related problems than the other age groups and than children from DNSGP-2.

**Table 3:** Comorbidity and number of daily health-related problems (according to parents) in children with CAH and control children from DNSGP-2

	**0-4** **CAH**	**0-4** **DNSGP-2**	**4-12** **CAH**	**5-12** **DNSGP-2**	**12-18** **CAH**	**13-18** **DNSGP-2**	**total** **CAH**	**total** **DNSGP-2**
	
chronic conditions *								
0	0.0	71.8	0.0	70.6	0.0	65.4	0.0	69.3
1	83.0	24.0	72.6	21.7	78.0	23.7	77.9	23.1
≥ 2	17.0	4.2	27.4	7.7	22.0	10.9	22.1	7.6
**Total**	**100.0**	**100.0**	**100.0**	**100.0**	**100.0**	**100.0**	**100.0**	**100.0**
**N**	**12**	**766**	**62**	**1494**	**32**	**874**	**106**	**3134**
**Number of daily health-related problems**								
0	42.0	17.0	26.0	20.6	31.0	8.7	33.0	15.4
1	16.6	20.9	6.0	21.9	16.0	16.4	12.9	19.7
2	8.0	19.3	18.0	16.9	19.0	15.2	15.0	17.1
3	16.6	14.9	10.0	12.5	12.0	14.3	12.9	13.9
≥ 4	16.6	27.9	40.0	28.1	22.0	45.4	26.2	33.8
**Total**	**100.0**	**100.0**	**100.0**	**100.0**	**100.0**	**100.0**	**100.0**	**100.0**
**N**	**12**	**766**	**62**	**1494**	**32**	**874**	**106**	**3134**

* chronic conditions, including CAH

Age boundaries differ slightly between CAH groups and DNSGP-2 groups.

### Experienced burden of the condition

Only 8% of the parents reported that their children experienced constraints in daily life as a result of CAH (Table 4). According to the parents, an adrenal crisis was experienced by 33% of the children. Most parents (83%) indicated that they did not fear the occurrence of an adrenal crisis. About 30% of children aged 4-18 have been absent of school due to CAH in the past year. The percentage of parents feeling their actions can influence the course of the condition declines when the age of the children increases. In total, 96% of the parents indicated to be satisfied with the overall health of their children. All adolescents were satisfied with their own health and 84% of them have no problem with controlling the condition (not shown in Table).

**Table 4:** Experienced burden of the condition (by parents)

	0-4 year		4-12 year		12-18 year		Total	
	
	n	*(%)*	n	*(%)*	n	*(%)*	n	*(%)*
Total	12	*(100)*	62	*(100)*	32	*(100)*	106	*(100)*
**Constraints in daily life** ((totally) agree)	0	*-*	6	*(10)*	2	*(6)*	8	*(8)*
**Adrenal crisis experienced **(yes)	3	*(25)*	20	*(32)*	12	*(38)*	35	*(33)*
**Fear for adrenal crisis** ((totally) agree)	3	*(25)*	15	*(24)*	0	*-*	18	*(17)*
**School absence due to CAH **(yes)	n/a	*-*	19	*(31)*	9	*(28)*	28	*(30)*
**Influence on condition** ((totally) agree)	9	*(75)*	38	*(61)*	19	*(59)*	66	*(62)*
**General health** (good/very good/excellent)	12	*(100)*	60	*(97)*	30	*(94)*	102	*(96)*

n/a = not applicable

Two scales have been constructed about parents' experiences (Table 5). The scale 'Experiences parents' contains items about parents' experience with the condition of their child (including their own ability to control the condition). The average score on this scale in all three age groups is slightly higher than 3, which indicates that parents are inclined to a more positive feeling. The scale 'Difficulties' contains items about the reasons why parents find it difficult to keep the condition of their child under control, including an item about their fear of an adrenal crisis. The average score on this scale is below 3 for all age groups. This means that on average, parents do not find it difficult to keep the condition of their child under control. The same holds true for the adolescents who have a mean score of 2.03 on the 'Difficulties' scale (not in Table).

**Table 5:** Parents' experience with the condition

	0-4 year	4-12 year	12-18 year
**Scale 'Experiences parents'**			
Average scale score (95% CI)	3.50 (3.06-3.94)	3.08 (2.92-3.25)	3.22 (2.94-3.50)
Cronbach's alpha	0.76	0.71	0.72
Number of items (range) 1-------------- 3 ------------5 Negative neutral positive	6 (1-5)	7 (1-5)	7 (1-5)
**Scale 'Difficulties'**			
Average scale score (95% CI)	2.18 (1.80-2.55)	2.27 (2.12-2.42)	1.82 (1.61-2.03)
Cronbach's alpha	0.78	0.73	0.85
Number of items (range) 1--------------3 ------------5 Less diff. neutral more diff.	8 (1-5)	8 (1-5)	8 (1-5)

### Management

About 6% of children older than 4 years do not use medication daily (Table 6). On the other hand, 82% of children use two or more different medicines for CAH, most often Hydrocortisone and Fludrocortisone. Approximately 88% of the parents report that a proper balance in medication use is achieved. Medication is administered at a set time by 83% of the parents. The percentage of parents that administer medication at fixed times declines when children get older. Of the adolescents, 17% (n = 4) indicate not to use any CAH related medication (not in Table). All of the children and adolescents that do not use medication (or, in case of the adolescents, say they do not use medication) have the classic form of CAH.

**Table 6:** Self management and Participation (according to parents)

	0-4 year		4-12 year		12-18 year		Total	
	
	n	*(%)*	n	*(%)*	n	*(%)*	n	*(%)*
Total	12	*(100)*	62	*(100)*	32	*(100)*	106	*(100)*

**Self management**								
**Daily medication use**								
0 CAH med.	0	*-*	4	*(6)*	2	*(6)*	6	*(6)*
1 CAH med.	0	*-*	7	*(11)*	6	*(19)*	13	*(12)*
2 CAH meds.	9	*(75)*	29	*(47)*	11	*(34)*	49	*(46)*
3 CAH meds.	3	*(25)*	22	*(36)*	13	*(41)*	38	*(36)*
Medication at fixed time	11	*(92)*	53	*(85)*	24	*(75)*	88	*(83)*
**Increased medication in past 12 months?**								
0 times	0	*-*	3	*(5)*	5	*(16)*	8	*(7.5)*
1-10 times	11	*(92)*	43	*(69)*	19	*(59)*	73	*(69)*
> 10 times	1	*(8)*	11	*(18)*	5	*(16)*	17	*(16)*
Unknown	0	*-*	5	*(8)*	3	*(9)*	8	*(7.5)*
**Participation**								
**Contact with school because of problems**	n/a	*-*	22	*(35)*	8	*(25)*	30	*(32)*
**Child exercises as much as peers without CAH**	n/a	*-*	41	*(66)*	22	*(69)*	63	*(67)*
**Child engages in a sport**	n/a	*-*	46	*(74)*	31	*(97)*	77	*(82)*

n/a = not applicable

Medication is most often raised in response to flu, fever, physical injury, and illness in general (Table 6). Parents of children younger than 12 years often mentioned inoculation or drawing blood as a reason to increase the medication whereas parents of children older than 12 years often mentioned exams as a reason to increase the medication.

#### Self-management

Almost 63% of children aged 4-12 take their medicines independently or with some support of their parents and 56% of children aged 12-18 take their medication independently, according to their parents. 63% of the adolescents indicate that they take their medication independently from their parents and 38% of those adolescents receive help from a parent or other family member. The mean age at which children start to self-administer medication is 7.6 years (CI: 6.81 - 8.48). Approximately 67% of parents of children aged 12-18 years check whether the medication has been taken correctly. Approximately 75% of the adolescents take their medication according to the instructions of their physician.

#### Preventive measures

The measures that children and their parents take to prevent an adrenal crisis were also investigated. Only 7 of 106 children were able to inject themselves glucocorticoids intramuscularly in case of an adrenal crisis. On average 59% of all children carry an SOS bracelet or badge, children aged 4-12 more often (66%) than older (59%) or younger (25%) children. More than half of the adolescents carry an SOS bracelet or badge. However, only 17% carry a crisis card or emergency injection. According to parents, 31% of children aged 12-18 always carry a crisis card or emergency injection with them. For the younger age groups, parents most often carry a crisis card or emergency injection with them. Most parents of children with CAH state that their children's friends are aware of the fact that their child has a chronic medical condition.

### Participation

As shown in Table 4 in the past 12 months, 30% of children have been absent from school for CAH related reasons. The average duration of school absence was approximately 7 days. 32% of the parents had contact with school because of school problems in the past 12 months. The reasons for contact varied from providing information and education to teachers to problems with bullying, behavioural problems and problems caused by comorbidity such as autism or dyslexia. With regard to physical exercise, 67% of the parents indicate that their child exercises just as much as peers without CAH (Table 6). Furthermore, 82% of children engage in a sport, often mentioned are horse riding, swimming, soccer and tennis. About 84% of parents of children older than 4 years indicate that their children have one or more hobbies. Playing outdoors, and playing computer games are often mentioned among children aged 4-12 and playing outdoors, meeting with friends, playing computer games and playing a musical instrument are often mentioned among children aged 12-18. About 28% of children older than 12 have a part time job next to school. On average they work 5.8 (+/-1.26) hours a week. According to their parents, none of these children experience problems at work as a consequence of CAH.

To evaluate the degree of protectiveness or even over-protectiveness that parents may display towards their children, some questions were asked only to parents of children of certain age groups. Firstly, parents of children aged 0-4 were asked if they make use of child care facilities and 75% indicated to do so. A playgroup or day nursery was mentioned most often. Secondly, parents of children aged 4-12 were asked whether their child sometimes spends the night away from home, for example, with family or friends. About 85% of parents reported that this sometimes occurred.

None of the adolescents indicated to experience impediments like concentration problems, physical discomfort or pain. When asked how they would rate their own level of functioning in daily life, all adolescents reported to be satisfied, with answers varying from good (35%) to very good (30%) and excellent (35%).

Finally, parents of children aged 12-18 were asked if their children go out by themselves or whether they go on holiday independent of their parents. Less than half of the children (41%) go out by themselves and 69% has occasionally been on holiday independent of their parents. Unfortunately, for these participation questions we have no comparative data available from the general population.

## Discussion

In this study, we evaluated the physical, social and societal functioning of children with CAH and their parents. Previous research has focused mainly on adult patients with Addison's disease and CAH so to our knowledge this study is the first describing health state and quality of life of children with CAH and their parents.

In general, our results show that children with CAH experience few negative CAH related effects and that they participate well in several areas such as school and leisure time. Adolescents are confident about the management of their condition and their participation in daily life. Parents do not seem to be exceptionally protective of their children and are capable of leaving the care of their child to others.

Depending on the tools used mixed results are found but previous studies in Norway [13,14], Germany [15,16] and the United Kingdom [8,17] have shown impaired quality of life in adult patients with Addison's disease and CAH. Particularly, reduced energy, vitality and general health perception were reported. The parents of children with CAH in our population, however, did not report any of these problems. Instead, most parents indicated they were satisfied with the general health of their child with CAH. Comorbidity was present in most adult CAH patients studied by Erichsen [13]. In contrast, in our population comorbidity was scarce. That may explain the absence of impaired quality of life in our group children. Recently, Arlt et al. described the health status of adult CAH patients in the United Kingdom [8] and showed that a minority of adult CAH patients in the United Kingdom are under endocrine specialist care and that androgen levels are often poorly controlled whereas children with CAH are managed using established guidelines. This may be another important factor in explaining the higher quality of life in children compared to adults. Our results show some discrepancy between the answers of parents of adolescents and adolescents themselves with regard to medication intake. 17% of adolescents indicate not to use any CAH related medication compared to only 6% indicated by their parents. This may be explained by the assumption that parents may not be aware of the fact that their child has stopped taking medication.

Our results show that as children grow older, parents become less afraid of an adrenal crisis. This is probably because parents get more familiar with the disease. When children grow older, the responsibility for the management shifts from parent to child, as is the case in all chronic conditions and healthy children. This transfer of responsibility and control is often difficult for parents as their role in managing the disease decreases [18]. We found that the increasing importance of self-management seems to pose few problems for children with CAH since they are partly capable of self-management already at a young age. However, measures to prevent adrenal crisis can be improved. Although CAH patients have to carry a card or SOS bracelet that indicates the use of glucocorticoids in case an emergency occurs, our results show that many children don't do so It is important to stress the necessity of such preventive measures.

A limitation of our study is a lack of a reference group of healthy children for all variables. Furthermore, we used questionnaires adapted from a previous Dutch study [16] that hinders comparisons to other studies.

Because group sizes were relatively small detailed statistical models could not be used.

## Conclusions

In conclusion, our study shows that quality of life is not reduced in children with CAH and their parents. The children do experience several daily health related problems but these do not hamper them in their daily activities and participation in society. Parents themselves do not experience a lot of fear about the occurrence of an adrenal crisis and feel that they can influence the condition of their child. However, measures for prevention of adrenal crisis could be improved.

## Competing interests

There are no potential conflicts of interest with respect to financial or personal relationships.

## Authors' contributions

SS and TW designed the questionnaires and conducted the study. SS drafted the manuscript and performed the statistical analysis. TW helped to draft the manuscript. HC and BO participated in the design of the questionnaires, participation of patients and revised the manuscript. All authors read and approved the final manuscript.
